# Oxaliplatin-Based Chemotherapy in Patients with Metastatic Colorectal Cancer Aged at Least 75 Years: A Post-Hoc Subgroup Analysis of Three Phase II Trials

**DOI:** 10.3390/cancers11040578

**Published:** 2019-04-24

**Authors:** Gerardo Rosati, Stefano Cordio, Giorgio Reggiardo, Giuseppe Aprile, Alfredo Butera, Antonio Avallone, Aniello Tucci, Giuseppe Novello, Giuseppina Blanco, Giuseppe Caputo, Domenico Bilancia, Roberto Bordonaro

**Affiliations:** 1 U.O. Oncologia Medica, Azienda Ospedaliera S. Carlo, 85100 Potenza, Italy; domenicobilancia@gmail.com; 2 Struttura Complessa di Oncologia Medica, Ospedale Garibaldi, 95122 Catania, Italy; cordiostefano@gmail.com (S.C.); oncoct@hotmail.com (R.B.); 3 Biostatistics and Data Management Unit, Medi Service S.r.l., 16149 Genova, Italy; giorgio.reggiardo@mediservice.it; 4 Dipartimento di Oncologia Clinica, Ospedale San Bortolo, AULSS8, 36100 Vicenza, Italy; giuseppe.aprile@aulss8.veneto.it; 5 U.O. Oncologia Medica, Ospedale S. Giovanni di Dio, 92100 Agrigento, Italy; butera.alfredo@tiscali.it; 6 Struttura Complessa di Oncologia Clinica Sperimentale Addome, Dipartimento Assistenziale e di Ricerca dei Percorsi Oncologici del Distretto Addominale, Istituto Nazionale per lo Studio e la Cura dei Tumori “Fondazione Giovanni Pascale” — IRCCS, 80131 Napoli, Italy; avalloantonio@gmail.com; 7 U.O. Oncologia Medica, ASL NA3 Sud, 80035 Nola (NA), Italy; aniello.tucci@tin.it; 8 U.O. Oncologia Medica, Azienda Ospedaliero-Universitaria “Vittorio Emanuele”, 95124 Catania, Italy; giuseppe.novello@virgilio.it; 9 U.O. Oncologia Medica, Istituto Oncologico del Mediterraneo, 95029 Viagrande (CT), Italy; giusiblanco@virgilio.it; 10 U.O. Oncologia Medica, Azienda Ospedaliera Gravina, 95041 Caltagirone, Italy; caputogs@tiscali.it

**Keywords:** colorectal cancer, doublet chemotherapy, elderly patients, oxaliplatin

## Abstract

Patients older than 75 years of age are usually excluded from metastatic colorectal cancer studies based on a combination chemotherapy containing oxaliplatin. Our group conducted three phase II trials in elderly patients in recent years. A post-hoc subgroup analysis of 67 patients aged at least 75 years was included in this study. Oxaliplatin was combined with capecitabine in two trials and with uracil-tegafur (UFT) plus folinic acid in the third trial. In one study, bevacizumab was also added to chemotherapy. The median age of patients was 77 years, and all had a good performance status (0 to 1). The observed overall response rate was 45%, comparable to younger patients (51%, *p* = 0.49). The estimated median progression-free survival (PFS) time and overall survival (OS) time were 8.7 and 19.3 months, respectively. These results did not significantly differ from those in younger patients (8.0 months for PFS (*p* = 0.58) and 19.7 months for OS (*p* = 0.94), respectively). The most common grade 3–4 adverse events included diarrhea (13%), fatigue (13%), peripheral neuropathy (10%), and neutropenia (7%). Moreover, the toxicity was never statistically different from that in younger patients. The efficacy of oxaliplatin-based combination was maintained in fit elderly patients ≥75 years.

## 1. Introduction

Colorectal cancer (CRC) is the third most common cancer in Western countries. As mortality from other non-cancer causes declines with a corresponding lengthening of the average life, CRC incidence has been increasing in older patients—so much so that it has a median age of onset of 71 years at present and more than 30% of patients with new diagnoses are aged at least 75 years [1,2]. Treating an 80-year-old patient with chemotherapy can be risky, and the optimal choice of treatment should be personalized [3]. Often, these subjects have impaired hepatic or renal function and a compromised bone marrow reserve. Yancik et al. analyzed this topic and verified that patients >75 years with CRC have a mean of five different comorbidities [4,5]. In addition, older people may be more focused on their quality of life (QoL) and on therapies that can lead to symptomatic improvement rather than cyclically undergoing chemotherapy which can be toxic, and which may cause health issues both to the patient and to family members [6]. Although the oncologist’s use of a monochemotherapy could reconcile the attempt to offer some advantage to these patients without altering their QoL, it is also true that—at least for patients defined as “fit”—the International Society of Geriatric Oncology (SIOG) guidelines recommend doublet chemotherapy [7]. In this context, a randomized phase II study showed that in elderly patients it is preferable to use an oxaliplatin-based chemotherapy doublet rather than irinotecan because it determines the same efficacy but reduces toxicity [8]. A retrospective analysis included 3742 CRC patients (614 aged ≥70) from four clinical trials and tested an oxaliplatin-based chemotherapy. This analysis showed that there were no differences between young and elderly patients in terms of efficacy and toxicity patterns, or in the doses of drugs administered. However, the authors pointed out that the patients enrolled in the trials were selected and the results were not reproducible for all elderly subjects. Furthermore, three of the four studies had treated patients up to 75 years of age [9]. Few studies, mostly retrospective and not particularly explicative as only a small number of subjects were analyzed, have evaluated the possibility that an oxaliplatin-based chemotherapy doublet can be beneficial for octogenarians without provoking an unsustainable increase in toxicity [10,11,12,13,14]. Based on these data, the aim of this analysis was to evaluate whether a cohort of patients aged at least 75 years and enrolled in three phase II studies (one of these randomized) comprising a population of elderly subjects had the same clinical benefits without increased toxicity from a combined oxaliplatin and fluoropyrimidine chemotherapy [8,15,16].

## 2. Results

### 2.1. Patient Characteristics

The study population consisted of 67 patients. Their characteristics are listed in Table 1. Their median age was 77 years (range: 75–89) and 16 patients were over 80 years old. All had a performance status (PS) between 0 and 1, and the majority of them had a single metastatic site, generally in the liver (58%). Adjuvant treatment was given to 30% of patients, chemotherapy to those with previous colon carcinoma, and chemoradiotherapy to those with previous carcinoma of the rectum. Sixty percent of the subjects had at least one comorbidity. The most frequent comorbidity was cardiovascular, which was reported in approximately 45% of patients. Respiratory and genitourinary diseases were diagnosed in more than one-fifth of the patients, while about 15% of the subjects had diabetes mellitus.

### 2.2. Treatment Cycles and Dose Reductions

Sixty-seven senior patients received 407 cycles with oxaliplatin, while those younger than 75 years received 458 cycles. The median number of cycles was six for all patients (range:1–12 for both groups). Following significant toxicity, a 20% reduction in chemotherapy doses was planned in 21 older patients. This was necessary in 27 patients <75 years, but the difference was not statistically significant (*p* = 0.41). As a result of unacceptable toxicity, we report treatment discontinuation in 10% of older patients. A similar rate of treatment withdrawal was noted in subjects <75 years (13%, *p* = 0.68), as described in Table 2.

### 2.3. Efficacy

**Figure 1:**
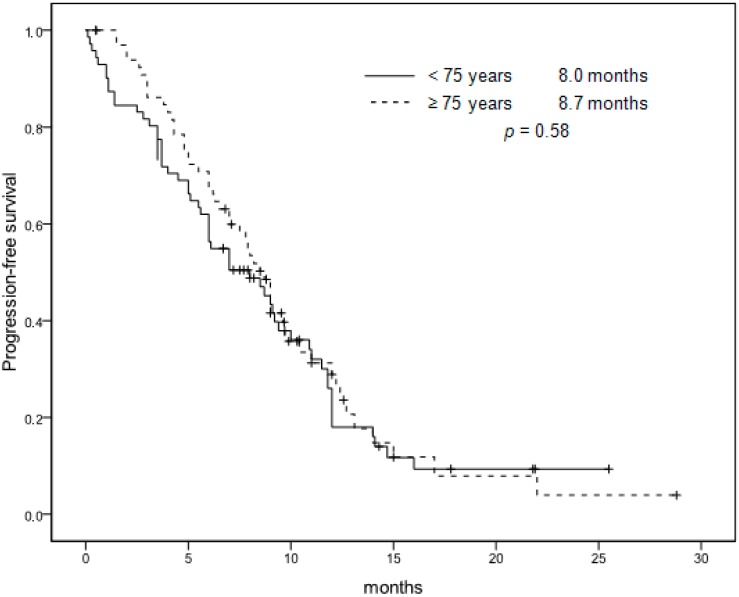
Progression-free survival. Patients <75 years versus patients ≥75 years.

**Figure 2:**
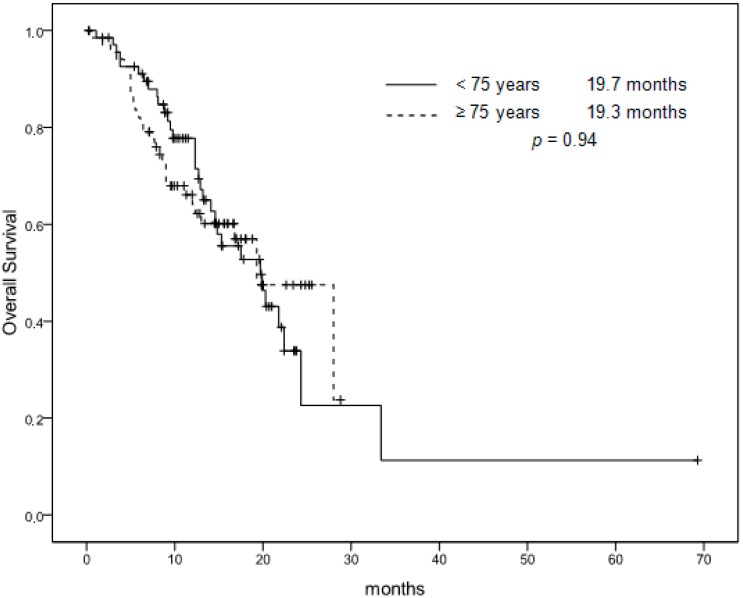
Overall survival. Patients <75 years versus patients ≥75 years.

All patients were assessable for response and all data in the two groups are shown in Table 3. The overall response rate (ORR: complete response (CR) + partial response (PR)) was 45% [95% confidence interval (CI), 32.5% to 57.0%]. In addition, 26 patients (39%) had a stable disease (SD), whereas a progressive disease (PD) was documented in 11 cases (16%). Response rates (RRs) of patients ≥75 years were comparable to younger patients (51% (95% CI, 38.8% to 62.6%), *p* = 0.49). Following response to chemotherapy, two patients (3%) in the first group and four patients (6%) <75 years underwent an attempt at curative liver metastasectomy, but this difference was not statistically significant (*p* = 0.45). The estimated median progression-free survival (PFS) time was 8.7 months (95% CI, 7.6 to 9.7 months), and median overall survival (OS) time was 19.3 months (95% CI, 13.8 to 24.7 months). As with tumor RR, PFS and OS did not differ from that reported in patients <75 years (respectively, 8.0 months, *p* = 0.58, and 19.7 months, *p* = 0.94) (Figure 1 and Figure 2). The analysis performed on the cohort of patients treated with bevacizumab (*n* = 27) confirms that this population was not statistically different (*p* = 0.36) from the total cohort of patients enrolled in the three studies (*n* = 111). Three patients underwent subsequent complementary locoregional treatment (radiotherapy for an inoperable pelvic recurrence of a rectal carcinoma, *n* = 1; stereotactic radiotherapy of pulmonary metastases, *n* = 2). Twenty-three patients (34%) received second-line therapy, in most cases an irinotecan-based regimen. Only four patients received third-line treatment. Similarly, 32% of patients younger than 75 years received second-line chemotherapy based on irinotecan. This difference was not statistically significant (*p* = 0.81).

### 2.4. Safety

All patients were evaluated for safety. The most frequent treatment-related grade 3–4 toxic effects are reported in Table 2. The main severe toxicities were diarrhea and fatigue/asthenia, both of which occurred in 13% of patients without statistically significant differences in the group of younger patients (17% for diarrhea and 11% for fatigue/asthenia, respectively). Peripheral neuropathy attributed to oxaliplatin was observed in a similar manner in each group (10% and 11%, respectively). The most frequent serious hematological toxicity was neutropenia, which was recorded in five older patients (7%) and in four (6%) among those younger than 75 years. Also, in this case, the differences were not statistically different (*p* = 0.66), similar to all the other severe toxicities following the therapy. Only two older patients were hospitalized for febrile neutropenia. However, tolerability was manageable, and no toxic death occurred in the group of older patients ≥75 years.

## 3. Discussion

Although the results of prospective phase II trials and/or retrospective analyses of elderly patients with metastatic CRC (mCRC) have already evaluated the opportunity of administering an oxaliplatin-based chemotherapy doublet [10,11,13,14,17,18], we believe we have contributed to clarifying some aspects in the subgroup of subjects aged 75 years or older. First of all, our results are equivalent not only in terms of activity and efficacy to those jointly analyzed in older patients aged less than 75 years, but surprisingly also in terms of feasibility and safety. Secondly, all patients had been evaluated prospectively in three previously published trials and the current comprehensive case study of 67 cases is among the most extensive series published on this topic. Chemotherapy is often prescribed and administered differently by clinicians in the geriatric population. Some authors found age to be amajor deterrent in the choice of chemotherapy for patients over 75 years of age [19,20]. Others pointed out that chemotherapy could also be refused by elderly patients in view of their decline in functional and mental status, but also because many of them prefer to safeguard their quality of life (QoL) to the detriment of potential clinical benefits, fearing drug toxicities [21,22]. Other authors have shown that if a potentially more toxic chemotherapy is chosen for these patients, it could be advisable to reduce drug dosages from the beginning of the treatment or to modify the schedule of the therapy by eliminating the administration of the bolus of 5-fluorouracil (5-FU) and/or modulation of folinic acid (FA), thus reducing toxicities [17,21,23,24]. Indeed, to prevent clinicians from offering chemotherapy to the elderly based only on their own experiences and acumen, validated tools such as the Comprehensive Geriatric Assessment (CGA) could provide an objective evaluation and assist oncologists in the decision-making process for elderly patients, distinguishing those who are fit from those who are frail [25]. It is therefore evident that a potentially toxic treatment such as an oxaliplatin-based chemotherapy doublet should be reserved only for the former highly selected patients. Unfortunately, when our three previous phase II trials were designed, the CGA had not yet been standardized and the elderly were selected through careful analysis of both their comorbidities and their PS. Although this may appear to be a limitation of the current analysis, Crosara Teixeira et al. have shown that an Eastern Cooperative Oncology Group (ECOG) PS of 0–1 was directly associated with greater survival of patients than those with an ECOG ≥2 [26]. Considering that the patients in our study had an ECOG PS of 0–1, the resulting efficacy is truly remarkable in this older population. On the other hand, multi-agent chemotherapy has long been considered the standard of first-line care for patients with mCRC, and a meta-analysis stated a greater RR, PFS, and OS benefit with upfront combination therapy [27]. With the advent of new targeted therapies, the median OS for all patients is approximately 30 months after diagnosis [28,29]. It might therefore seem that the median OS of 19.3 months recorded in our cohort is lower, but this was due not only to the fact that only a third of the patients had received second-line therapy and even fewer received third-line therapy, but also because only 27 of them received an upfront therapy with bevacizumab together with chemotherapy. However, Landre et al. demonstrated in a meta-analysis that doublet chemotherapy does not provide an OS benefit compared to single-agent 5-FU in elderly patients over 75 years of age and it is widely evident that the elderly can obtain a survival benefit from simpler regimens, as in the AVEX trial [30,31]. Our cohort reported an ORR of 45%, two patients (3%) underwent an attempt at curative liver metastasectomy, and their median PFS was 8.7 months. These data are in line with those reported in the literature and show that an initially more aggressive therapeutic strategy could be of particular benefit in some categories of patients, especially those with limited liver metastases who are potentially resectable [24,30]. Even if the postoperative mortality rate is significantly higher among elderly compared to younger patients, two large studies have reported that liver resections seem safe and could offer a substantial advantage in terms of overall disease-free interval in patients older than 70 years [32,33]. In our series, the toxicity profile was very favorable, with only 7% and 13% patients developing grade 3–4 neutropenia and diarrhea or fatigue/asthenia, respectively. Severe sensory neuropathy occurred in only 10% of patients, regardless of age. There were no statistically significant differences for all the other severe adverse events following the therapy compared to the group of younger patients. The absence of statistically significant differences between the two groups of patients was also appreciable relative to the percentages of patients who had discontinued treatment for unacceptable toxicity. Although this is undoubtedly due to the selection of patients, the contained toxicity is related to the combination of oxaliplatin with an oral fluoropyrimidine compared to 5-FU, as already demonstrated in the literature [34].

## 4. Patients and Methods

The three phase II trials that were the object of our data extrapolation had enrolled a total of 185 patients aged ≥70 years: (1) Ninety-four patients randomized to CAPOX (*n*= 47) received oxaliplatin 65 mg/m^2^/day on days 1 and 8 as an intravenous (i.v.) infusion and oral capecitabine 1000 mg/m^2^ twice daily for 14 consecutive days, every 3 weeks, while those randomized to CAPIRI (*n*= 47) received irinotecan 80 mg/m^2^/day on days 1 and 8 as an i.v. infusion combined with capecitabine as previously described, every 3 weeks; (2) Forty-seven subjects treated with oxaliplatin 65 mg/m^2^/day on days 1 and 8 i.v. plus oral uracil/tegafur (UFT) 300 mg/m^2^/day and oral FA 90 mg/day for 14 consecutive days, every 3 weeks; (3) Forty-four patients treated with oxaliplatin 130 mg/m^2^ i.v. on day 1, oral capecitabine 1000 mg/m^2^ twice daily for 14 consecutive days, plus bevacizumab 7.5 mg/kg i.v. on day 1, every 3 weeks. A detailed description of the methods of patient selection, inclusion and exclusion criteria, methods of dose reduction in relation to the reported toxicities, radiological procedures adopted for the evaluation of efficacy, methods considered for evaluating the toxicity, and statistical considerations are reported in the three papers considered [8,15,16]. The principal characteristics of patients were: histologically confirmed metastatic or locally advanced CRC; bidimensionally measurable disease; age of 70 years or more; ECOG PS predominantly between 0 and 1; life expectancy of ≥3 months; adequate bone marrow, kidney, and liver function; no previous chemotherapy for advanced disease; no serious illness or medical condition. Therapy continued until disease progression, unacceptable toxicity, or patient refusal, except in the study where continuous administration of bevacizumab was given once every 3 weeks and chemotherapy was repeated up to a maximum of 8 cycles. At the time of disease progression, patients could receive second-line treatment according to the investigator’s free choice. The main objectives of the studies were to evaluate the efficacy and safety of the combinations of therapies used, while the secondary objectives were the PFS, the OS, and the assessment of the QoL through the use of the European Organization for Research and Treatment of Cancer (EORTC) questionnaire (QLQ-C30). All analyses were carried out on an intention-to-treat basis. In the randomized study, the Data Service Center provided for stratification by institution, sex, ECOG baseline PS (0 versus 1), site of primary disease (colon versus rectum), and number of metastatic sites (1 versus ≥1) to ensure that these prognostic factors were well balanced between the two proposed chemotherapy schemes (CAPOX versus CAPIRI). Median OS and PFS were calculated using the Kaplan–Meier method and differences between the levels of possible prognostic factors were compared using the log rank test in univariate analyses [35]. A value of *p* < 0.05 was considered statistically significant. Statistical analyses were performed using SPSS version 21.0 (SPSS Inc., Chicago, IL, USA). An optimal two-stage design was used to determine the number of patients to be included in each individual study [36]. Written informed consent was obtained from patients before enrolment, and all proceedings were conducted in accordance with the Declaration of Helsinki (2008). The three studies were approved by the institutional review board of the San Carlo Hospital, Potenza, Italy (IRB approval number: 2002-148, 2004-001159-12 and 2010-019463-10, respectively).

## 5. Conclusions

Our study shows that age alone should not be considered as an absolute contraindication to the use of a more intensive chemotherapy schedule (doublets plus or minus biologics) in the treatment of patients aged over 75 with mCRC. Nevertheless, the use of a chemotherapy doublet comprising oxaliplatin in this subcategory of elderly patients should be restricted to carefully selected cases with optimal performance status. In this context, only a careful geriatric assessment will allow us to opt for the most appropriate treatment for each individual patient.

## Figures and Tables

**Table 1:** Patient distribution, ≥75 years.

Characteristics	Oxaliplatin Plus UFT/FA (*n* = 21)	Oxaliplatin Plus Capecitabine (*n* = 27)	Oxaliplatin/Capecitabine Plus Bevacizumab (*n* = 19)	All (*n* = 67) (%)
Median age (range)	76 (75–89)	78 (75–85)	78 (75–83)	77 (75–89)
Male/female	12/9	15/12	11/8	38/29 (57/43)
ECOG PS 0/1	10/11	16/11	14/5	40/27 (60/40)
Colon/rectum	13/8	16/11	14/5	43/24 (64/36)
Liver/lung/other sites	11/4/6	14/7/6	14/3/2	39/14/14 (58/21/21)
Involved sites ≥ 1	11/10	11/16	11/8	33/34 (49/51)
Adjuvant therapy	7	9	4	20 (30)
Comorbidity	11	17	12	40 (60)

ECOG PS = Eastern Cooperative Oncology Group Performance Status; FA: folinic acid; UFT: uracil/tegafur.

**Table 2:** Frequency of chemotherapy intolerance and grade 3–4 toxicity per patient (%).

Characteristics	<75 Years	≥75 Years	*p* Value
Diarrhea	12 (17%)	9 (13%)	0.57
Stomatitis	2 (3%)	2 (3%)	0.95
Nausea/vomiting	3 (4%)	2 (3%)	0.70
Peripheral neuropathy	8 (11%)	7 (10%)	0.88
Laryngeal spasm	3 (4%)	2 (3%)	0.70
Fatigue/asthenia	8 (11%)	9 (13%)	0.70
Fever/chills	3 (4%)	4 (6%)	0.64
Hyperbilirubinemia	2 (3%)	1 (1%)	0.59
Anemia	3 (4%)	3 (4%)	0.94
Hand-foot syndrome	3 (4%)	4 (6%)	0.64
Neutropenia	4 (6%)	5 (7%)	0.66
Thrombocytopenia	4 (6%)	3 (4%)	0.76
20% dose reduction	27 (38%)	21 (31%)	0.41
Early discontinuation	9 (13%)	7 (10%)	0.68

**Table 3:** Comparison of elderly patients (≥75 years) with younger patients (<75 years).

	≥75 Years, *n* = 67	<75 Years, *n* = 71	*p* Value
Response rate	30 (45%)	36 (51%)	0.49
Median PFS	8.7 months	8.0 months	0.58
Median OS	19.3 months	19.7 months	0.94
Additional therapeutic lines	23 (34%)	23 (32%)	0.81
Liver metastasectomy	2 (3%)	4 (6%)	0.45

PFS: progression-free survival; OS: overall survival.
